# Percussion hemoglobinuria - a novel term for hand trauma-induced mechanical hemolysis: a case report

**DOI:** 10.1186/1752-1947-5-508

**Published:** 2011-10-07

**Authors:** Monica Vasudev, Barbara A Bresnahan, Eric P Cohen, Parameswaran N Hari, Sundaram Hariharan, Brahm S Vasudev

**Affiliations:** 1 Division of Nephrology, Medical College of Wisconsin, 9200 W Wisconsin Avenue, Milwaukee WI 53226, USA; 2 Division of Allergy and Clinical Immunology, Medical College of Wisconsin, 9000 W Wisconsin Avenue, Milwaukee, WI 53226, USA; 3 Division of Hematology and Oncology, Medical College of Wisconsin, 9200 W Wisconsin Avenue, Milwaukee, WI 53226, USA

## Abstract

**Introduction:**

Extracorpuscular hemolysis caused by mechanical trauma has been well described in relation to lower extremity use, such as in soldiers and runners. Terms such as "march hemoglobinuria", "foot strike hemolysis" and "runners hemoglobinuria" have previously been coined and are easily recalled. Newer cases, however, are being identified in individuals vigorously using their upper extremities, such as drum players who use their hands to strike the instrument. Given the increased recognition of upper extremity-related mechanical hemolysis and hemoglobinuria in drummers, and the use of hand drumming worldwide, we would like introduce a novel term for this condition and call it "percussion hemoglobinuria".

**Case presentation:**

A 24-year-old Caucasian man presented with reddish brown discoloration of his urine after playing the djembe drum. Urine examination after a rigorous practice session revealed blood on the dipstick, and 0 to 2 red blood cells per high power field microscopically. The urine sample was negative for myoglobulin. Other causes of hemolysis and hematuria were excluded and cessation of drum playing resulted in resolution of his symptoms.

**Conclusions:**

The association of mechanical trauma-induced hemoglobinuria and playing hand percussion instruments is increasingly being recognized. We, however, feel that the true prevalence is higher than what has been previously recorded in the literature. By coining the term "percussion hemoglobinuria" we hope to raise the awareness of screening for upper extremity trauma-induced mechanical hemolysis in the evaluation of a patient with hemoglobinuria.

## Introduction

Extracorpuscular hemolysis due to mechanical trauma was originally described using the term "march hemoglobinuria" by Fleischer in 1881, in a young soldier following a strenuous field march [1]. Since then, hemoglobinuria has been described in both genders and after a wide range of activities. It has been associated with walking, running and marching [2], and also with Japanese fencing and karate [3]. A few authors have also described hemoglobinuria following African drum playing [4,5]. Tobel *et al*. characterized 45 healthy individuals who participated in a cultural hand drumming event in Uruguay, and confirmed extracorpuscular hemolysis as a cause of rust urine [6]. Factors which influence hemoglobinuria was described by Davidson in 1969, who demonstrated that the individual running style, type of foot wear and the running surface were independent variables, and modification of these could prevent hemoglobinuria [7]. A reduction in hemolytic episodes by use of rubber insoles in shoes or protective covering over hands has also been noted [8].

Typically, patients with extracorpuscular hemolysis due to mechanical trauma present with reddish brown urine in the setting of increased serum indirect bilirubin and lactate dehydrogenase, and decreased serum haptoglobin. The absence of myoglobin in the urine confirms hemoglobinura.

Terms such as "foot strike hemolysis" and "runners hemoglobinuria" have been coined and are easily recalled by medical students, house staff and practitioners. Given the increased recognition of upper extremity-related mechanical hemolysis and hemoglobinuria in drummers, we would like introduce a novel term for this condition called "percussion hemoglobinuria".

## Case presentation

**Figure 1:**
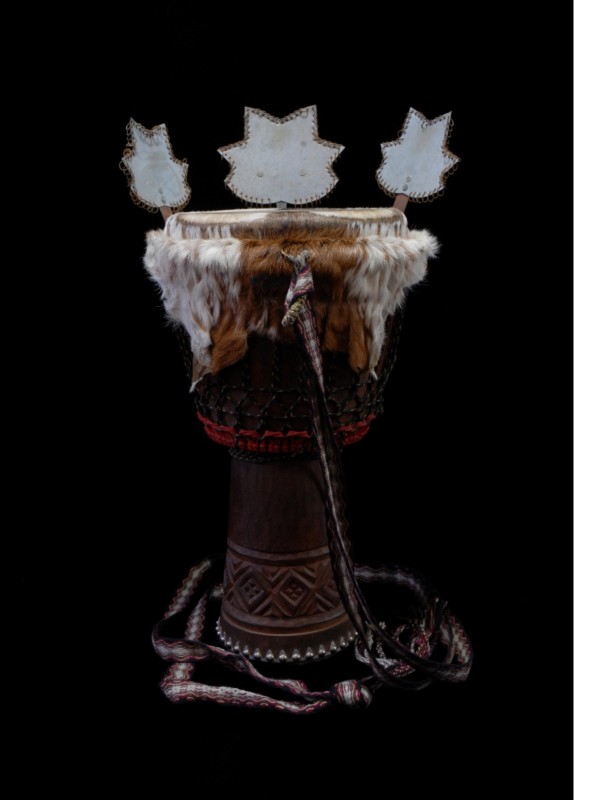
**Djembe Drum**.

A previously well 24-year-old Caucasian man was evaluated for a six-month history of episodes of passing dark colored urine. Each episode typically followed a djembe drum playing session (Figure 1). Despite playing these drums for many years, he was playing them with increased frequency and duration over the past six months since joining a new djembe drum circle. He would typically play these drums intensely for two hours at a time. He used the palm of his hands to percuss and of late observed his hands to be bruised after each session. Recently, he also noticed blisters on his fingers and thumbs with the formation of calluses.

His urine turned reddish brown in color, the intensity of which correlated to the duration and intensity of his drum playing. His urine returned to normal color within 12 to 24 hours. He had associated myalgia, and on two occasions experienced back pain. Activities such as heavy weightlifting, or working out on a treadmill did not change the color of his urine nor did these changes in urine color occur in association with a viral upper respiratory tract infection. He denied flank pain, abdominal pain, dysuria, frothy urine or passing stones in his urine. He denied fever, easy bruisability, jaundice, prior blood transfusion, skin rash, itching or angioedema.

He had an unremarkable past medical or surgical history, and he was not taking any medications. He denied any family history of renal disease or hematuria. He drank alcohol and smoked cigarettes socially. He denied any active drug abuse.

On physical examination, he was afebrile with a blood pressure of 122/72 mmHg, a pulse rate of 68 beats/min and a body mass index of 24 kg/m^2^. A physical examination was remarkable for a lack of costovertebral angle tenderness and peripheral edema. He had multiple calluses on the palmar aspect of both thumbs and palms.

His pre-drum playing serum chemistry, complete blood count, reticulocyte count and RBC (red blood cell) osmotic fragility test were normal. His urine was clear, and urine analysis was normal. On urine microscopy he had occasional granular casts but no RBCs or cellular casts. His 24-hour urine analysis revealed a protein excretion of 165 mg/24 hr without microalbuminuria and his creatinine clearance was normal. Abdominal imaging was pursued for his complaint of back pain and was negative for nephrolithiasis. His kidney size was normal without anatomical abnormalities.

Post-drum playing, his urine sample was reddish brown in color with a specific gravity of 1.030, pH 5.5, 1+ protein, 3+ blood, 0 to 2 RBC/high powered field (HPF) and 0 to 2 white blood cells/HPF with negative nitrite and leukocyte esterase. His urine microscopy revealed 1 to 2 RBC/HPF. His urine was negative for myoglobin and his serum creatine kinase was mildly elevated at 407 mg/dL. Plasma haptoglobin and lactate dehydrogenase measured 12 hours post-exercise was normal.

Our patient informed us of other members who had similar discolored urine following strenuous drum playing. In the words of our patient, "in the culture of drum players, if the urine does not become dark after a drum playing session, one has not played hard enough".

His myalgia, back pain and reddish urine resolved when he abstained from vigorous participation in his djembe drum circle.

## Discussion

This case illustrates transient traumatic intravascular hemolysis secondary to percussion using the palms. Few case reports in the past have described this phenomenon. We propose to name this phenomenon "percussion hemoglobinuria" given the fact that this occurrence is common in passionate hand percussion drum players, as observed by our patient, as well as in prior documented cases in the literature. Concomitant nonsteroidal anti-inflammatory drugs or cocaine use, dehydration and hemoglobinuria can potentially predispose drummers to acute renal failure [5,9,10].

Percussion hemoglobinuria is very similar in pathogenesis to march hemoglobinuria, originally described by Fleischer in 1881 [1], except that it occurs in drum players and the intravascular hemolysis occurs in the palms instead of soles. Djembe drums have their origin in West Africa (Figure 1). They stand approximately 24 inches tall, 12 to 14 inches at the widest diameter, are goblet shaped and usually covered in goat skin. They are meant to be played with bare hands. Different tones are produced depending on the technique used to strike the skin. Producing these tones requires forceful percussion as experienced by the authors (BSV, MV).

Various mechanisms of hemoglobinuria have been proposed. In 1881, Fleischer suggested that a primary hematological disorder was responsible for hemoglobinuria [1] but Dickinson argued in 1894 that the hemoglobinuria was probably a response to physiological stress [11]. The repetitive mechanical trauma caused to the sole or palm results in injury to the RBCs, causing release of hemoglobin into the intravascular space. Once all available binding sites on haptoglobin are saturated, the free hemoglobin is subsequently filtered by the kidney to produce hemoglobinuria and hence the dark urine. Hemoglobinuria may not occur in all drum players globally, taking into account factors such as protective recoil from the earth's floor, or using a neck strap to suspend the drum [4].

Case reports of glutathione peroxidase deficiency leading to excessive hemolysis [12], low plasma haptoglobin levels leading to hemoglobinuria [13] and erythrocyte membrane-protein anomaly in march hemoglobinuria have been described [14]. Based on electrophoretic patterns of RBC from three patients demonstrating stress-induced hemolysis [15], an erythrocyte membrane abnormality, which could lead to increased susceptibility to hemolysis, has also been suggested.

## Conclusions

The diagnosis of percussion hemoglobinuria should be considered in drum players who have repeated trauma to their palms followed by dark urination. Evaluation should include excluding other causes of hemolysis, including testing for RBC fragility secondary to a membrane or enzyme defect. Myoglobinuria should be excluded. Protective covering for the hands should be offered. Our patient informed us about other people in his drum circle who have dark urine after strenuous drum playing. We hypothesize that this entity is more prevalent than reported in the literature. The long-term effects of chronic hemoglobinuria in professional drum players have yet to be explored.

## Consent

Written informed consent was obtained from the patient for publication of this case report and any accompanying images. A copy of the written consent is available for review by the Editor-in-Chief of this journal.

## Competing interests

The authors declare that they have no competing interests.

## Authors' contributions

BV evaluated our patient and followed up on appropriate tests. He co-wrote the manuscript. MV helped diagnose the case and co-wrote the manuscript. PH provided a hematology consult to exclude hematological causes of dark urine. EPC helped diagnose the case and reviewed the manuscript. SH helped diagnose the case and reviewed the manuscript. BAB helped diagnose the case and reviewed the manuscript. All authors read and approved the final manuscript.
